# Small airway resistance in obese and nonobese patients with obstructive sleep apnea syndrome using impulse oscillometry

**DOI:** 10.55730/1300-0144.5809

**Published:** 2024-01-05

**Authors:** Nejdiye GÜNGÖRDÜ, Aytan İSMAYİLOVA, Nigar ALİYEVA, Tamer A M ALHELOU, Ayşenur ÖZDİL ESER, Ilgım VARDALOĞLU KOYUNCU, Nihal ENŞEN, Ersan ATAHAN, Şermin BÖREKÇİ, Bilun GEMİCİOĞLU

**Affiliations:** 1 Department of Pulmonary Diseases, Cerrahpaşa Faculty of Medicine, İstanbul University-Cerrahpaşa, İstanbul, Turkiye; 2 Department of Public Health, Cerrahpaşa Faculty of Medicine, İstanbul University-Cerrahpaşa, İstanbul, Turkiye

**Keywords:** Obstructive sleep apnea syndrome, impulse oscillometry, airway resistance

## Abstract

**Background/aim:**

There is limited information on the pathologic changes in the small airways among obese and nonobese patients with obstructive sleep apnea syndrome (OSAS). Impulse oscillometry (IOS) measures airway resistance and reactance independently of patient effort. This study aimed to compare airway resistance in small airways using IOS between obese and nonobese patients with OSAS.

**Materials and methods:**

In this real-life cross-sectional study, demographic information was collected from obese and nonobese subjects diagnosed with moderate and severe OSAS without any other underlying diseases. Spirometry and IOS measurements were conducted, and the values of both groups were statistically analyzed.

**Results:**

The nonobese group had a mean age of 45.6 ± 11.7 years (median 45), while the obese group had a mean age of 48.4 ± 9.5 years (median 47.5). The mean body mass index (BMI) for the nonobese group was 26.2 ± 2.1 kg/m^2^ (median 27 kg/m^2^), and for the obese group, it was 35.6 ± 6.4 kg/m^2^ (median 33 kg/m^2^). Statistically significant differences were observed between the two groups in R5 - R20 percentage, reactance area (AX), and resonant frequency (Fres) values (p < 0.05).

**Conclusion:**

Among obese OSAS patients, there is an increase in resistance in small airways as indicated by IOS values. IOS shows promise as a potential screening tool for diagnosing OSAS.

## 1. Introduction

Obstructive sleep apnea syndrome (OSAS) is characterized by recurrent episodes of complete (apnea) or partial (hypopnea) upper airway obstruction during sleep, often accompanied by decreased blood oxygen saturation [1]. Disease severity is gauged by the apnea-hypopnea index (AHI), which measures the frequency of sleep-disordered breathing episodes per hour during sleep. Sleep-related breathing disorders can lead to significant nocturnal hypoxemia and permanent damage to the neurocognitive and cardiovascular systems. Symptoms of sleep apnea include snoring, diagnosed apnea, choking, morning headache, fatigue, sleepiness, and an increased risk of cardiovascular and metabolic diseases such as hypertension, coronary artery disease, heart failure, arrhythmia, stroke, diabetes, and insulin resistance. Obesity is one of the most recognized risk factors for OSAS. Studies indicate that the risk of OSAS increases 8–10 times in individuals with a body mass index (BMI) exceeding 29. Alongside obesity, factors such as increased tongue base, uvula width, and neck circumference contribute to OSAS predisposition. A neck circumference exceeding 43 cm in men and 38 cm in women is considered a significant risk factor for OSAS. Furthermore, while weight gain exacerbates existing OSAS symptoms, weight loss has been shown to ameliorate the condition [2–6].

Oscillometry serves as a noninvasive measurement method for assessing respiratory functions in clinical settings. Impulse oscillometry (IOS) stands out as a practical and time-efficient technique, independent of respiratory effort, thanks to its ability to measure the reactance and resistance of airways, encompassing both large and small airways. Among IOS parameters, Resistance (R) signifies airway resistance, while Reactance (X) denotes airway conductance. R5, measured at 5 Hertz (Hz) sound waves, reflects total airway resistance, while X5 indicates reactance. R20 indicates resistance at 20 Hz, primarily representing resistance in large airways. Difference between R5 and R20 (R5 - R20) proves to be a sensitive parameter in demonstrating small airway resistance [7–9]. Increased airway resistance and elevated R5 values due to nasal obstruction are more prevalent in OSAS, positively correlating with the AHI and negatively correlating with the lowest oxygen saturation [10]. OSAS patients often exhibit heightened total airway resistance due to narrow upper airways [11]. Another study on airway resistance in OSAS patients revealed that they had higher airway resistance in the supine position than in the upright position, indicating a large difference in airway resistance when changing from the upright to the supine position, whereas this feature was not observed in normal individuals [12,13]. Such findings suggest potential abnormalities in the upper airway structure among OSAS patients. Specifically, OSAS patients tend to have smaller pharyngeal airways, prone to collapse due to their unique shape, and more susceptible to narrowing in the supine position. These characteristics may contribute to heightened upper airway resistance [14–16].

It has been noted that resistance and reactance tend to rise with increasing BMI among normal subjects [17]. Hence, it is plausible to hypothesize that both disease and obesity could exert a bidirectional influence on OSAS patients. However, there is a scarcity of information regarding pathological alterations in the small airways among obese and nonobese OSAS patients. This study aimed to demonstrate that IOS has the capability to identify pathological changes in all airways, particularly in the small airways, among both obese and nonobese individuals with OSAS.

## 2. Materials and methods

The study protocol was approved by the Ethics Committee of the Medical Faculty of İstanbul University-Cerrahpaşa (Date: 05.01.2023, No. 564371) and adhered to the ethical standards outlined in a suitable edition of the Declaration of Helsinki. Written informed consent was obtained from all participating patients.

### 2.1. Study design and setting

The study was conducted as a cross-sectional investigation from January 6, 2023, to June 6, 2023. It involved 60 patients, both obese and nonobese, diagnosed with moderate and/or severe OSAS through polysomnography. The research took place at the Chest Diseases Sleep Outpatient Clinic of Cerrahpaşa Medical Faculty.

### 2.2. Participants

Inclusion criteria:

- Patients aged between 18 and 65 years who presented to the Sleep Outpatient Clinic of the Department of Chest Diseases and were diagnosed with moderate or severe OSAS by polysomnography
- Individuals who provided consent by signing the informed consent form

Exclusion criteria:

- Individuals who did not provide consent by declining to sign the informed consent form.
- Patients diagnosed with mild OSAS.
- Patients without OSAS diagnosis.

### 2.3. Data sources/measurements

A data collection form was developed to assess the age, sex, comorbidities, and medications of each patient. In addition to physical examination, measurements of neck circumference, weight, and height were taken. Epworth Sleepiness Scale (ESS) scores, pulmonary function test (PFT) parameters (spirometry), impulse oscillometry measurements, and data from polysomnography (PSG) were all documented.

The minimum requirements for PSG are based on the recording protocol outlined in the American Association of Sleep Medicine (AASM) 2007 report. PSG was performed using the SOMNOscreen plus system (SOMNOmedics GmbH, Randersacker, Germany). Measurements were obtained and recorded over a period of 5–8 h during nocturnal sleep. PSG encompassed multiple parameters including electroencephalogram (EEG) (C3/A2, C4/A1, Fp1/A1, Fp2/A2, O1/A1, O2/A2), electrooculogram (EOG) (right and left), chin electromyogram (EMG) and EMG in two legs, ECG, nasal cannula, thermistor, tracheal microphone, body position, oximetry, and respiratory effort channels. The PSG results for each patient were scored by the same individual adhering to established standards. The scoring criteria outlined in the AASM 2012 guidelines were applied. Hypopneas were scored according to the AASM 2013 recommended criteria, requiring a ≥3% decline in oxygen saturation accompanied by a ≥30% decline in the amplitude of nasal airflow. The AASM’s third edition of the International Classification of Sleep Disorders delineates the clinical and sleep testing criteria for OSAS [18]. The severity of OSAS can be classified according to the number of respiratory events observed per hour, termed the AHI: mild OSAS (AHI: 5–14.9 events per hour), moderate OSAS (AHI: 15–29.9 events per hour), and severe OSAS (AHI >30 events per hour) [19]. Additionally, the oxygen desaturation index (ODI) was calculated as the number of oxygen desaturations per hour during total sleep time, with a 3% desaturation threshold utilized.

The patients’ height (cm), weight (kg), and BMI (kg/m^2^) were recorded. Obesity classification for those diagnosed with moderate and severe OSAS was determined based on BMI (in kilograms per square meter (kg/m^2^). BMI categories were defined as nonobese (<30 kg/m^2^) and obese (≥30 kg/m^2^) [20].

Spirometry test results including forced vital capacity (FVC), FVC%, forced expiratory volume in one second (FEV1), FEV1%, FEV1/FVC, and FEV1/FVC% were recorded. These tests were conducted using a ZAN 100 Flow Handy II device in accordance with the criteria outlined by the European Respiratory Society and American Thoracic Society (ERS/ATS) [21].

IOS (Vyasis Jaeger) measurements were conducted with subjects seated while a technician supported their cheeks and they wore nose clips, adhering to standard recommendations [22]. Briefly, subjects were instructed to tightly seal their lips around the mouthpiece and breathe quietly at functional residual capacity levels. Impulse signals generated at 0.2-s intervals. The rectangular pressure impulses are superimposed on airflow and fed to the airway through a mouthpiece during tidal breathing following confirmation of stable spontaneous volume and airflow. A minimum of three consecutive measurements lasting >30 seconds were taken. The device measured values including resistance (R), reactance (X) at frequencies of 5 Hz, 10 Hz, 15 Hz, and 20 Hz, impedance (Z), resonance frequency (Fres), and reactance area (AX). Resistance at 5 Hz (R5) gives information about the resistance of the whole airway, while R20 provides information about the resistance in large airways, and R5-R20 gives information about the resistance in small airways. Reactance indicates the airways’ ability to expand against pressure. Impedance (Z) is the sum of resistance and reactance. For IOS measurements, we used respiratory impedance (Zrs) at 5 Hz (Zrs5) and mean whole-breath values of respiratory resistance (Rrs) and reactance (Xrs) between 5 Hz and 20 Hz in 5 Hz increments (R5–R20 and X5–X20, respectively), and resonant frequency [22].

### 2.4. Study size

From January 6, 2023, to June 6, 2023, a total of 210 patients suspected of having OSAS underwent polysomnography. Among them, OSAS was not detected in 47 patients, and 41 patients with mild OSAS were excluded from the study. Additionally, 25 patients were outside the specified age range were excluded. Of the 97 patients invited to participate, 65 agreed. However, 5 patients were excluded due to inability to perform spirometry according to the required standards. Finally, the study comprised 60 patients, with 28 classified as obese and 32 as nonobese, on whom evaluations were conducted.

### 2.5. Statistical analysis

Statistical analyses were performed using the SPSS version 21.0 software package. Numerical variables were reported as mean ± standard deviation, median (interquartile range), while categorical variables were presented as frequency and percentage. The conformity of numerical variables to normal distribution was assessed visually (histogram, Q-Q graph) and analytically (Shapiro–Wilk test). The Pearson chi-square test was used to compare categorical variables. A value of p < 0.05 was accepted as the level of statistical significance. For pairwise comparisons of continuous numerical variables, the independent sample t-test was used for normally distributed data, while the Mann–Whitney U test for nonnormally distributed data. Statistical significance was set at p < 0.05.

## 3. Results

In this study, a total of 60 OSAS patients, comprising 18 (30%) women and 42 (70%) men, were evaluated. The mean age of the participants was 46.9 ± 10.8 years. The obese group comprised 28 cases. The mean age of the nonobese group was 45.6 ± 11.7 years (median 45), while the mean age of the obese group was 48.4 ± 9.5 years (median 47.5). There was no statistically significant difference between obese and nonobese patients in terms of age and smoking status (p > 0.05). Among patients with a history of metabolic disease, 65% (n = 13) were obese, whereas 37.5% (n = 15) of patients without metabolic disease were obese, and this difference was statistically significant (p = 0.044) (Table 1).

Comparisons of PFT and IOS results between obese and nonobese participants are presented in Table 2. R5, R5 - R20, R5 - R20 percentage, AX, and Fres values were significantly higher in the obese group compared to the nonobese group (p = 0.004, p = 0.029, p = 0.005, p = 0.005, p = 0.005, and p = 0.017, respectively). However, there were no statistically significant differences between the groups in terms of other parameters (p > 0.05) (Table 2).

**Figure 1:**
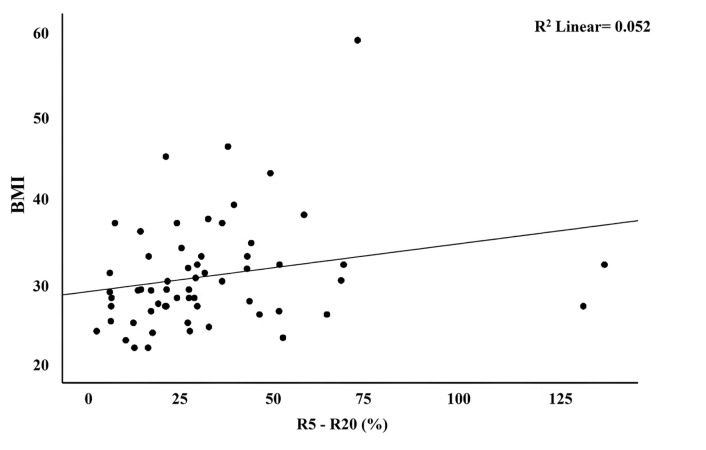
The relationship between BMI and R5 - R20 (%) values of the participants

**Figure 2:**
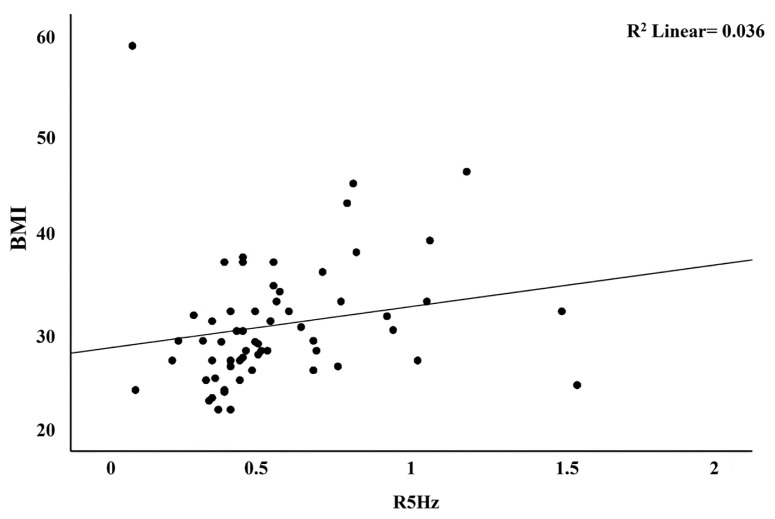
The relationship between participants’ BMI and R5 (Hz) values.

There was a positive correlation between BMI and R5 - R20 (%) and R5 values (rs = 0.317, rs = 0.416; p = 0.014, p = 0.001, respectively) (Figures 1 and 2).

There was no statistically significant correlation between R5 - R20, R5 - R20 (%), X5, X5 (%), AX, Fres parameters and AHI, ODI, RDI (p > 0.05) (Table 3).

## 4. Discussion

In our study designed to evaluate the variance in small airway resistance using IOS among obese and nonobese OSAS patients, we observed that the percentage of R5 - R20, AX, and Fres values were higher in the obese group. This suggests a heightened small airway resistance in obese OSAS patients. Consequently, our study reinforces the efficacy of IOS in detecting such differences. Notably, no spirometry dysfunction was detected in our participants; however, an early increase in respiratory system resistance was found in the obese group.

The changes in the respiratory system of obese individuals may be closely related to the excess adipose tissue surrounding the chest wall, abdomen, and upper respiratory tract, potentially leading to a reduction in lung volume and structural changes that affect respiratory system resistance [23,24]. While Brazzalle et al. emphasized the importance of spirometry evaluation in confirming obstructive changes in the respiratory physiology of obese individuals, Melo et al. demonstrated that a significant proportion of obese individuals are prone to developing a restrictive pattern in respiratory function. Interestingly, some studies have reported normal spirometry results in obese patients [23–26]. In our study, spirometry values for both obese and nonobese patients with OSAS were within the normal range, and there was no significant difference between the groups in terms of pulmonary function test parameters.

In addition to diagnosing and monitoring diseases directly associated with bronchial obstruction, impulse oscillometry (IOS) may prove valuable in assessing patients with OSAS. Oscillometric variables assessing total respiratory resistance (R5) and peripheral airway resistance (R5 - R20) appear to offer greater sensitivity than spirometry in detecting airway obstruction [9,27]. Given its capability to detect upper airway stenosis or patency in OSAS patients, IOS holds potential as a screening tool for OSAS diagnosis. In one study, R5 was notably higher and Xrs lower in OSAS patients compared to the control group. Moreover, the study revealed a significant correlation between the X5 parameter and disease severity, as defined by the AHI and functional residual capacity on body plethysmography, with the latter being lower in obese individuals [28]. Another study demonstrated that X5 in the supine position emerged as the most effective method for predicting OSAS, exhibiting a sensitivity of 73% and a specificity of 84% at an optimal cutoff point of 2.3 [8]. In a study involving three small patient groups (OSAS, COPD, and control), the R20 value was significantly higher in OSAS patients compared to those with COPD and the control group. However, the R5 - R20 value was higher in COPD patients than in the OSAS group, and in turn, higher in the OSAS group than in the control group [11]. Aihara et al. reported an independent positive correlation between proximal airway resistance (R20) in IOS and AHI in obese male OSAS patients [29]. These studies suggest that R20, associated with proximal airway resistance, can be used in evaluating patients with OSAS. Other studies observed an increase in total and peripheral resistance parameters with an increase in OSAS severity in both male and female patients. Significant increases in both of these resistance parameters were particularly evident in obese subjects with OSAS compared to obese subjects without OSAS. These abnormalities may stem from a pathologic increase in lung elasticity during expiration, which is also associated with a decrease in lung volume [30]. Kostrzewska et al. compared IOS with spirometry and found that the sensitivity of R5 - R20 was higher than the FEV1/FVC ratio in detecting airway obstruction in OSAS patients [31]. In a study conducted in China, researchers found higher respiratory resistance levels at 5 Hz (R5) and 20 Hz (R20) in severe OSAS patients and stated that respiratory resistance increased in large airways and there was a compensatory decrease in small airway resistance in OSAS patients [32]. In our study, no significant differences were found between the groups regarding pulmonary function test parameters. However, significant increases were noted in the R5 - R20 percentage, AX, and Fres values in IOS measurements of participants in the obese group. Additionally, a weak positive correlation was observed between BMI and R5 - R20 values in the participants. One limitation of this research is that it was conducted on subjects attending a sleep clinic at a general hospital, which may not be representative of the general population. Another limitation is the absence of a healthy control group, and lung volumes were not measured.

In conclusion, our findings indicate that the percentage of R5 - R20, AX, and Fres values were higher in the obese group, suggesting that small airway resistance is higher in obese OSAS patients. This suggests that obesity may exacerbate respiratory resistance and impair the function of OSAS patients. Additionally, IOS parameters can detect upper airway stenosis or patency in OSAS patients.

## Figures and Tables

**Table 1:** Sociodemographic characteristics of the participants.

	Total (n = 60)	Not obese (n = 32)	Obese (n = 28)	p
**Sex (n, %)**				**0.042** 1
Male	42 (70)	26 (61.9)	16 (38.1)	
Female	18 (30)	6 (33.3)	12 (66.7)	
**Age** Mean ± SD (Median)	46.9 ± 10.8 (46.5)	45.6 ± 11.7 (45)	48.4 ± 9.5 (47.5)	0.313^2^
**Smoking habits (n, %)**				0.597^1^
Smoking	16 (26.7)	7 (43.8)	9 (56.3)	
Nonsmoker	26 (43.3)	14 (53.8)	12 (46.2)	
Smoking cessation	18 (30)	11 (61.1)	7 (38.9)	
**Cigarettes** (pack/year). Mean ± SD (Median)	12.6 ± 10.9 (12)	12.7 ± 11.2 (10.5)	12.4 ± 10.8 (12)	0.952^3^
**Medical history (n, %)**				
No comorbidities	22 (36.7)	14 (63.6)	8 (36.4)	0.224^1^
Metabolic disease	20 (33.3)	7 (35)	13 (65)	**0.044** 1
COPD	9 (15)	4 (44.4)	5 (55.6)	0.721^4^
Cardiovascular disease	15 (25)	8 (53.3)	7 (46.7)	1^1^
Other	9 (15)	5 (55.6)	4 (44.4)	1^4^

1 Pearson chi-square test;

2 Independent groups t-test;

3 Mann–Whitney U test;

4 Fisher’s exact test;

COPD, chronic obstructive pulmonary disease. Bold font indicates statistical significance.

**Table 2:** Comparison of PFT and IOS results of obese and nonobese participants.

Parameters	Nonobese (n = 32)	Obese (n = 28)	p

**FVC (L)**			0.211^1^

Mean ± SD	4.40 ± 1.25	3.98 ± 1.28	

Median (IQR)	4.23 (3.80–5.16)	4.10 (2.78–4.86)	

**FVC (%)**			0.678^2^

Mean ± SD	104.16 ± 20.47	104.25 ± 17.15	

Median (IQR)	106 (92.25–119.50)	103.5 (95.25–111.75)	

**FEV1 (L)**			0.481^2^

Mean ± SD	3.46 ± 1.05	3.36 ± 1.35	

Median (IQR)	3.45 (2.90–4.16)	3.25 (2.33–4.11)	

**FEV1 (%)**			0.411^2^

Mean ± SD	98.78 ± 21.42	95.25 ± 27.07	

Median (IQR)	104 (91–110.75)	98.50 (86.25–109.50)	

**FEV1/FVC %**			0.594^2^

Mean ± SD	77.67 ± 8.25	78.53 ± 8.49	

Median (IQR)	78.74 (71.42–83.86)	80.31 (75.47–82.48)	

**MEF 25-75 (L/s)**			0.841^1^

Mean ± SD	3.15 ± 1.40	3.08 ± 1.45	

Median (IQR)	2.92 (2.15–4.16)	3.03 (1.91–4.30)	

**FEV3 (L)**			0.213^1^

Mean ± SD	4.11 ± 1.33	3.69 ± 1.24	

Median (IQR)	3.98 (3.42–4.80)	3.79 (2.61–4.50)	

**FEV6 (L)**			0.878^1^

Mean ± SD	3.77 ± 1.47	3.83 ± 1.45	

Median (IQR)	3.88 (2.98–4.57)	3.97 (2.84–4.79)	

**R5 (kPa/L/s)**			
Mean ± SD	0.50 ± 0.27	0.72 ± 0.32	**0.004** 2
Median (IQR)	0.45 (0.38–0.52)	0.71 (0.50–0.93)	

**R5 (%)**			0.078^2^

Mean ± SD	173.13 ± 74.46	207.86 ± 89.76	

Median (IQR)	158.50 (131.50–180.75)	181 (148–282.75)	

**R20 (kPa/L/s)**			
Mean ± SD	0.38 ± 0.19	0.36 ± 0.15	0.632^1^
Median (IQR)	0.39 (0.26–0.51)	0.33 (0.24–0.47)	

**R20 (%)**			0.296^2^

Mean ± SD	160.59 ± 58.93	180.39 ± 67.59	

Median (IQR)	150.5 (130.5–176.25)	163.5 (132–223.75)	

**R5 - R20 (kPa/L/s)**			
Mean ± SD	0.15 ± 0.27	0.34 ± 0.64	**0.029** 2
Median (IQR)	0.07 (0.05–0.13)	0.14 (0.11–0.29)	

**R5 - R20 (%)**			**0.005** 2

Mean ± SD	25.93 ± 24.60	38.63 ± 26.18	

Median (IQR)	20.16 (11.95–28.49)	33.52 (23.51–47.16)	

**AX (kPa/L)**			**0.005** 2

Mean ± SD	2.01 ± 6.24	3.28 ± 9.47	

Median (IQR)	0.35 (0.18–1.07)	1.05 (0.50–2.56)	

**X5 (kPa/L/s)**			
Mean ± SD	−0.05 ± 0.06	−0.10 ± 0.12	0.228^2^
Median (IQR)	−0.03 (−0.06 to 0.02)	−0.08 (−0.14 to −0.03)	

**X5 (%)**			0.9^2^

Mean ± SD	−924.34 ± 15169.15	238.53 ± 2033.27	

Median (IQR)	83 (−618.50 to 1339.75)	89.50 (−436 to 454.75)	

**Fres (1/s)**			**0.017** 1

Mean ± SD	16.87 ± 7.45	21.24 ± 6.21	

Median (IQR)	16.71 (11.66–19.78)	21.05 (16.89–25.19)	

1 Independent groups t-test;

2 Mann–Whitney U test;

PFT, pulmonary function test; IOS, impulse oscillometry; R5, resistance at an oscillating frequency of 5 Hz; R20, resistance at an oscillating frequency of 20 Hz; R5 – R20, difference between resistance at 5 and 20 Hz; X5, reactance at an oscillating frequency of 5 Hz; AX, area under the reactance curve from 5 Hz to the resonant frequency; Fres, resonant frequency

**Table 3:** The relationship between AX, R5 - R20, X5, Fres parameters and AHI, ODI, RDI.

		AHI	ODI	RDI
**AX**	Correlation coefficient	0.089	0.030	0.085
	p	0.497	0.821	0.519
**R5 - R20 (%)**	Correlation coefficient	−0.026	−0.094	−0.037
	p	0.843	0.473	0.781
**R5 - R20**	Correlation coefficient	0.126	0.112	0.128
	p	0.342	0.398	0.335
**X5**	Correlation coefficient	0.072	0.108	0.074
	p	0.587	0.414	0.577
**X5 (%)**	Correlation coefficient	−0.128	−0.121	−0.124
	p	0.330	0.359	0.344
**Fres**	Correlation coefficient	0.179	0.104	0.173
	p	0.172	0.430	0.187

*Spearman correlation test;

AHI, apnea hypopnea index; ODI, oxygen desaturation index; RDI, respiratory disturbance index; R5 – R20, difference between resistance at 5 and 20 Hz; X5, reactance at an oscillating frequency of 5 Hz; AX, area under the reactance curve from 5 Hz to the resonant frequency; Fres, resonant frequency.
